# Geno2proteo, a Tool for Batch Retrieval of DNA and Protein Sequences from Any Genomic or Protein Regions

**DOI:** 10.1515/jib-2018-0090

**Published:** 2019-07-13

**Authors:** Yaoyong Li, Elisa Aguilar-Martinez, Andrew D. Sharrocks

**Affiliations:** School of Biological Sciences, Faculty of Biology, Medicine and Health, University of Manchester, Manchester M13 9PT, UK

**Keywords:** Genomics, Proteomics, Mapping between genome and proteome, DNA sequences, Protein sequences

## Abstract

The interconversion of sequences that constitute the genome and the proteome is becoming increasingly important due to the generation of large amounts of DNA sequence data. Following mapping of DNA segments to the genome, one fundamentally important task is to find the amino acid sequences which are coded within a list of genomic sections. Conversely, given a series of protein segments, an important task is to find the genomic loci which code for a list of protein regions. To perform these tasks on a region by region basis is extremely laborious when a large number of regions are being studied. We have therefore implemented an R package geno2proteo which performs the two mapping tasks and subsequent sequence retrieval in a batch fashion. In order to make the tool more accessible to users, we have created a web interface of the R package which allows the users to perform the mapping tasks by going to the web page http://sharrocksresources.manchester.ac.uk/tofigaps and using the web service.

## 1 Introduction

We now have the complete genome sequences of many organisms including humans which act as reference datasets for other genome-wide studies. For example, ChIP-seq studies uncover genomic regions bound by particular proteins whereas genome sequencing efforts are identifying DNA sequence variants associated with disease. Following alignment to the genome sequence, both of these approaches return lists of genomic region coordinates. However obtaining the underlying nucleotide sequences and their protein coding potential is not trivial. Similarly, given a list of protein regions, a biologist may need the corresponding genomic locations coding these protein regions in order to understand the genomic context of these protein regions. As the technology in genomics and proteomics advances quickly, more and more molecular biological studies will need the interconversion of genomic loci and protein regions. Here we develop a new package, geno2proteo, to address this issue.

Currently, finding the protein sequence of a coding region can be done by using the two web sites, UCSC genome browser [1] and Ensembl [2]. However, their capabilities of finding protein sequences of coding genomic regions are restricted in several ways. A manual procedure has to be implemented to find the protein sequences for a single genomic locus which must be repeated for any additional genomic loci, which becomes very time-consuming if the user has many genomic locations to work on. One can obtain the whole protein sequence encoded by a protein-coding transcript from the Ensembl web site. However, it is not straightforward to obtain the amino acid sequence of any genomic coding region from the Ensembl web site or database. The recently developed R Bioconductor package ensembldb [3] has the functionality for performing mapping between genomic coordinates and protein coordinates.

Note that other software are available for two related bioinformatic tasks; namely obtaining the DNA sequences of any genomic regions and the amino acid sequences of any protein regions. The R package Bsgenome [4], the toolkit BEDTools [5], and the online tool the UCSC Table Browser [6] can be used for obtaining the DNA sequences of any genomic regions. The web site UniProt [7] can be used for obtaining the protein sequences of any protein regions. The Python package Biopython [8] can perform both tasks. Another related software is BLAST (https://blast.ncbi.nlm.nih.gov/Blast.cgi), which finds regions of DNA or protein sequences which are significantly similar to the given sequences. It can also search protein sequences using a nucleotide sequence and vice versa. However, BLAST addresses a very different problem from the one solved by ensembldb and the package developed here, geno2proteo. The input of BLAST is a DNA or protein sequence itself and BLAST tries to find all the sequences in a genome or proteome database which are significantly similar to the input sequence. In contrast, the input of both geno2proteo and ensembldb is a genomic or protein region specified by the coordinates, and the output is the DNA and protein sequences which are an exact match for the input region.

The R package geno2proteo presented in this paper implements the two-way mapping between genome and proteome; namely, given a genome and the gene annotations, it finds the amino acid sequences coded by any given genomic regions and finds the genomic regions coding for any given protein regions. Moreover, geno2proteo performs these tasks in a batch fashion, namely it finds and generates an output file of the genomic coordinates or protein sequences of any number of genomic or protein regions from a single input file containing a list of genomic or protein regions. As a by-product, the R package geno2proteo also provides functions for two more tasks; namely obtaining the DNA sequences of any genomic regions and the amino acid sequences of any protein regions. An additional deliverable of our research was the creation of a web service based on the R package to allow the users who are not familiar with the R programming to perform the four genomic and proteomic tasks by simply going to the website http://sharrocksresources.manchester.ac.uk/tofigaps and using the online tool.

A summary of the comparison of our geno2proteo package with other public software or web services on performing the four genomic and proteomic tasks is presented in Table 1. The table also compares the range of species and strains that those tools can process.

**Table 1:** Comparing geno2proteo with other public software on the four tasks. Tasks are shown in the first four columns headings (bold) whereas the table content indicates the capability of each of the indicated software packages on each task.

	DNA sequences of genomic regions	Protein sequences of genomic regions	Genomic loci of protein regions	Protein sequences of protein regions	Species	Is it a web service
BSgenome [3], BEDTools [4]	A list of any genomic regions				Any user-defined	No
UCSC Table Browser [5]	A list of any genomic regions				only database species	Yes
UniProt [6]				A list of protein IDs	only database species	Yes
Biopython [7]	A list of any genomic regions			A list of any protein regions	Any user-defined	No
ensembldb [8]	A list of any genomic regions	A list of any genomic regions	A list of any protein regions	A list of any protein regions	Only database species	No
geno2proteo	A list of any genomic regions	A list of any genomic regions	A list of any protein regions	A list of any protein regions	Any user-defined	No
ToFiGAPS	A list of any genomic regions	A list of any genomic regions	A list of any protein regions	A list of any protein regions	human and mouse	Yes

## 2 Implementation

### 2.1 The R Package Geno2proteo

Given a specific genome and one version of its gene annotations, the R package geno2proteo exploits the exonic structure of the protein-coding transcripts contained in the gene annotations for the genome and proteome mapping tasks. It needs three external data files:

1. DNA sequences: a text file in FASTA format containing the DNA sequence of the genome that the user wants to use for analysing the data. It can be compressed by GNU Zip.
2. Gene annotations: a text file in GTF format containing the gene annotations of the same version of the genome of the same species as that of the DNA sequence file described above. It can also be compressed by GNU Zip.
3. Genetic coding table: a text file containing a genetic coding table of the codons and the amino acids coded by those codons.

The first two data files correspond to the specific genome and its gene annotations which one wants to use. The third file is used for the translation from DNA sequences to protein sequence. A file for the standard genetic coding scheme is provided in the package. The DNA sequence and gene annotation files can be downloaded from the Ensembl web site [9] and can be used directly by the functions in the package. Alternatively the user can create their own data files, as long as they are in the file formats required by the package, which will be useful if the user needs to use some data files which are not available from the public databases like Ensembl. The exact file format of those data files and how to download or create them are described in detail in the package’s user guide (Supplementary file 1).

**Figure 1:**
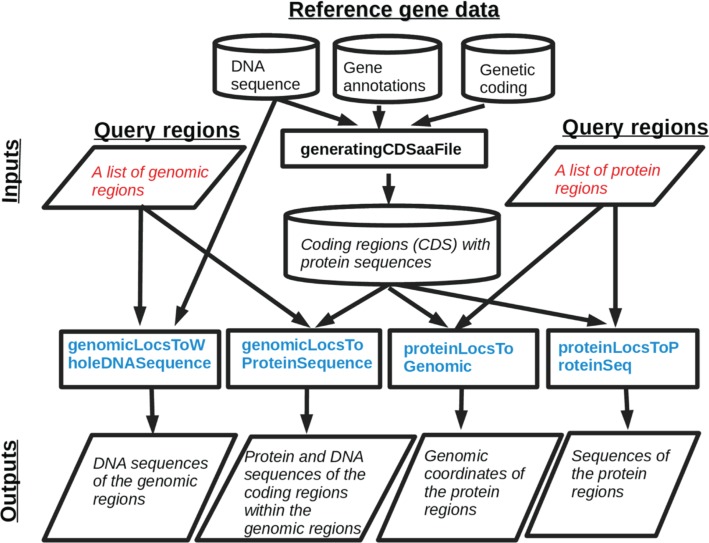
A workflow depiction of all the functions and their inputs, outputs and relations in the R package geno2proteo.

The geno2proteo package workflow starts by generating an internal data file, containing all the coding regions and their protein sequences, by using the three external reference data files and the R function *generatingCDSaaFile* provided in this package (Figure 1). Note that this internal file needs to be generated only once for one specific genome and one version of its gene annotations, which will be used subsequently by all the genome and proteome mapping tasks for the genome with this version of the gene annotations. The package has four main functions for the four tasks of obtaining the DNA and amino acid sequences of any list of genomic or protein regions, as depicted in the lower part of Figure 1, which are:

1. *genomicLocsToProteinSequence* takes a list of genomic regions as the input and finds the amino acid sequences and the DNA sequences of the coding regions within those genomic regions.
2. *genomicLocsToWholeDNASequence* takes a list of genomic regions and finds the whole DNA sequences of those genomic regions.
3. *proteinLocsToGenomic* finds the genomic regions coding for a list of protein regions.
4. *proteinLocsToProteinSeq* finds the amino acid sequences themselves for a list of regions in proteins specified by the coordinates of the regions along the proteins.

A more detailed explanation of all the functions in the package and how to use them are given in the package’s user guide (Supplementary file 1). The geno2proteo package is available in the R package CRAN repository, https://cran.r-project.org/package=geno2proteo.

### 2.2 The online tool ToFiGAPS

**Figure 2:**
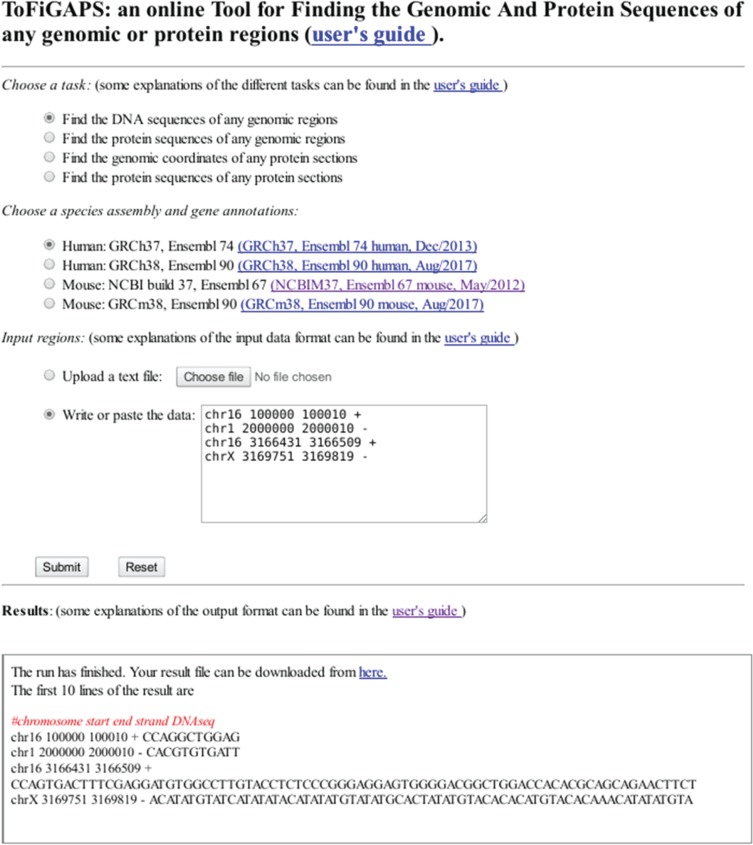
The main webpage of the online tool ToFiGAPS. This web interface performs the four genomic and proteomic mapping tasks. The web page address is http://sharrocksresources.manchester.ac.uk/tofigaps. The results box shows the DNA sequences retrieved from the selected human genome GRCh37 for the 4 genomic regions in the input box after clicking the button “Submit”, as the task “Find the DNA sequences of any genomic regions” was selected.

Based on the R package geno2proteo, we created a web service to give the users a more direct and simple way to use the functions provided in the R package. The user interface of the tool is in one single web page, as shown in Figure 2. To use the web service, simply go to the web page:

http://sharrocksresources.manchester.ac.uk/tofigaps

and follow the three simple steps:

Step 1: Choose one of the four tasks which one wants to perform.
Step 2: Choose a species genome and a gene annotation version.
Step 3: Input a list of genomic regions or proteomic regions.

Then click the Submit button, and after waiting a short time, the results will be shown in the results box in the bottom part of the web page. A detailed explanations about how to use the tool and the formats of the input and output are in the User’s Guide of the online tool, which can be accessed from the tool’s main web page. Currently two species, human and mouse, with two versions of genome for each of them are available in the web site. If the user wants to use other versions of human or mouse genome or any other species’ genome, he/she will have to use the R package geno2proteo which provides the function to deal with any species (for details see the section about the R package geno2proteo).

## 3 Application

**Figure 3:**
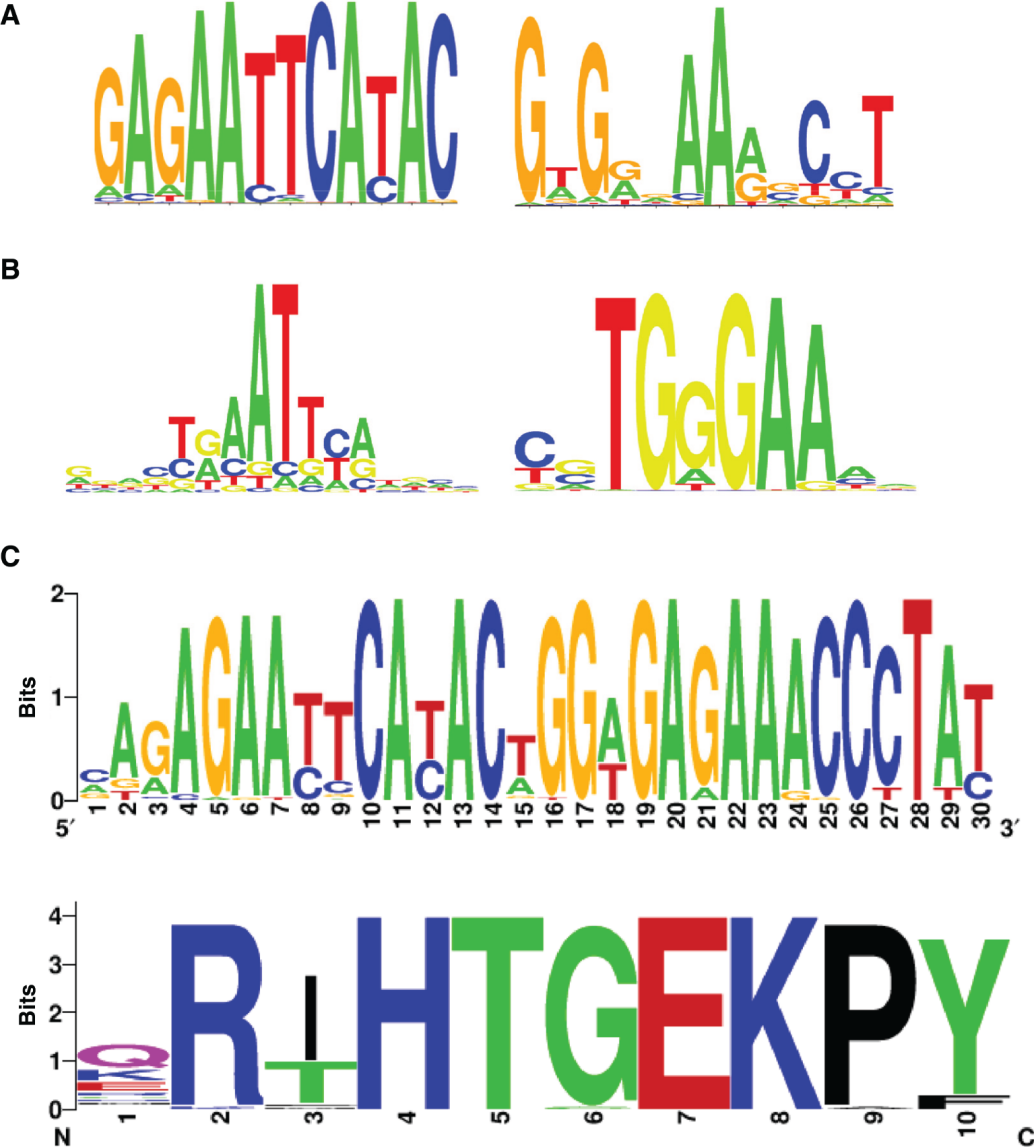
Motif analysis of the SUMO binding regions. (A) Two de novo motifs uncovered by motif discovery using HOMER [14] in SUMO2/3 ChIP-seq data, visualised by using WebLogo [15]. The left motif is similar to SOX18’s binding motif, and the right motif is similar to RBPJ1’s binding motif. (B) SOX18 and RBPJ1 binding motifs which were identified by HOMER as the known motifs being similar to the two de novo motifs in (A), created by Seq2Logo [16]. (C) The logo graphs of the DNA (top) and protein (bottom) sequences associated with the 44 “SOX18-2bp-RBPJ1” motif matching sites found in the SUMO ChIP-seq data. Two more nucleotides in the up- and down-stream of the matching sites were also included, because they belong to the same DNA translation frame as the first and last nucleotide within the matching sites according to the genes in which they are located.

Protein modification with small ubiquitin-like modifier (SUMO) plays an important regulatory role on the activities of hundreds of proteins associated with various biological functions [10]. For example, it can enhance the repressive activities of transcriptional regulators and does so by a myriad of mechanisms, including enhancing co-repressor recruitment [10]. To further study how SUMO might impact on gene regulation, we generated the SUMO2/3 ChIP-seq data from the MCF10A cell line to determine the genome-wide SUMO2/3 binding sites in these cells. MCF10A cells were treated with 1.8 ng/ml epidermal growth factor (EGF) for 30 min and one sample was generated using an anti-SUMO2 antibody (Life Technologies) according to previously described protocol [11]. The reads were aligned to the human genome hg19 using the software Bowtie 2 [12], and then 28,663 SUMO-associated regions (i.e. peaks) were identified from the aligned reads using the software MACS2 [13]. The ChIP-seq data is publicly available from ArrayExpress with the accession number E-MTAB-7759. Upon visual inspection of the data, we noticed that a large number of SUMO peaks were found near to transcriptional termination sites. We therefore selected all SUMO-associated regions located within +/−2 kb of a transcriptional termination site (n = 329) and performed motif enrichment analysis using the software Homer [14] to identify potential common binding motifs that might hint at a particular DNA binding protein. We found that the two most enriched novel DNA motifs are similar to the binding motifs of SOX18 and RBPJ1, respectively (Figure 3A). Note that the motifs shown in Figure 3A are not the binding motifs of SOX18 and RBPJ1 themselves as shown in Figure 3B. Instead they are the two de novo motifs that the software Homer uncovered from the 329 selected SUMO peaks to which the SOX18 and RBPJ1 motifs are the most similar known motifs according to the software Homer. We found 44 regions where the matching sites of the two motifs are close to each other and have the “SOX18 motif” up-stream of the “RBPJ1 motif” with a 2 bp gap between them (the genomic coordinates of those 44 regions in hg19 are in Supplementary file 2). We also found that all of these 44 regions are within the protein coding regions of genes encoding zinc finger proteins. We therefore asked whether there was an underlying DNA sequence motif or whether this was an indirect consequence of a highly conserved amino acid sequence giving rise to nucleotide sequence conservation due to the underlying common codon usage. We therefore needed the protein sequences of these multiple genomic regions, which was the original motivation for us to create the software presented in this paper.

**Figure 4:**
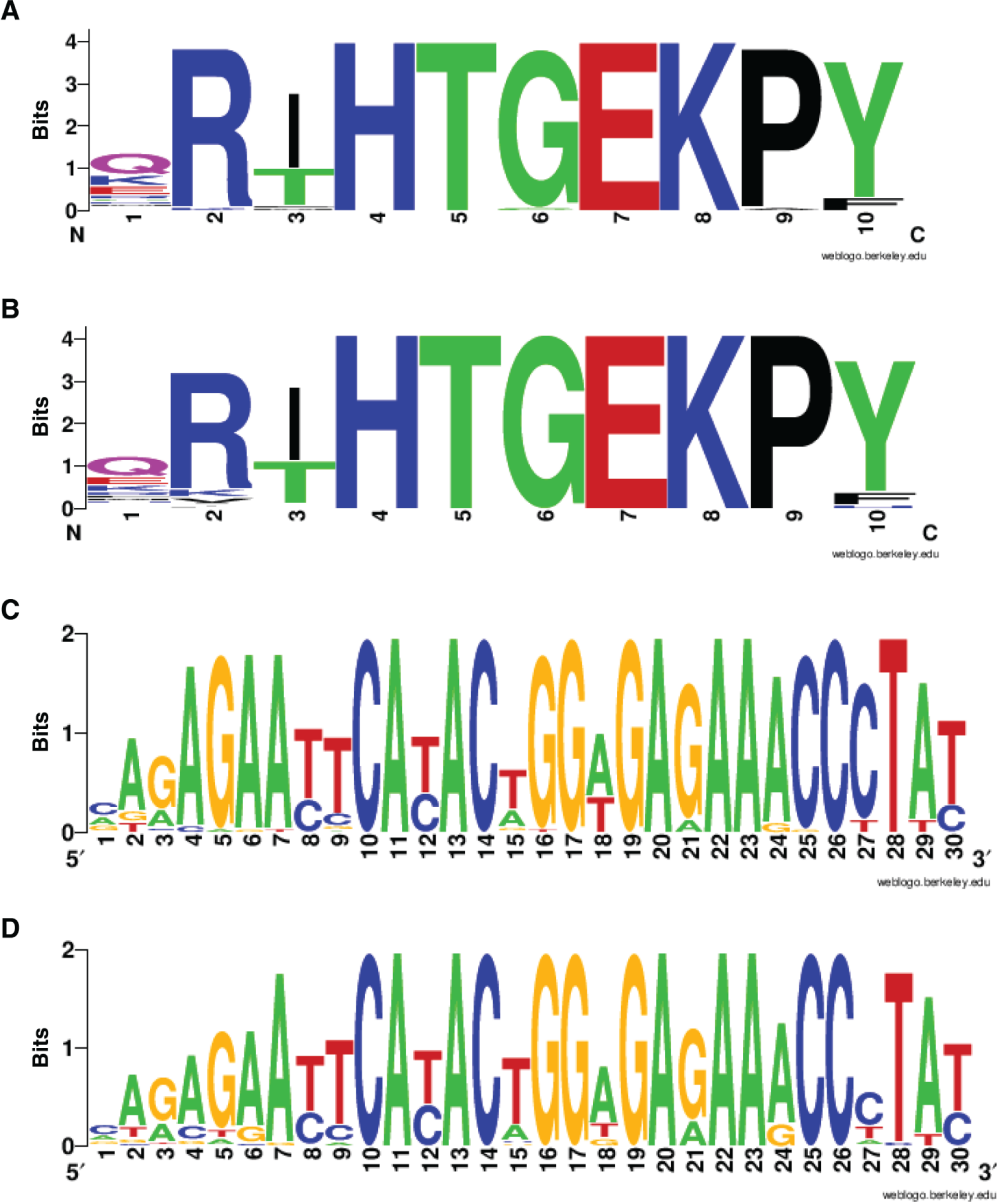
Comparison of the sequence motifs in adjacent repeated protein regions. The amino acid and DNA sequences of the 44 selected motifs associated with SUMO binding (A and C), are compared to the equivalent motifs on the same proteins/genes, in the immediate N-terminal or C-terminal regions (B and D). These motifs have similar amino acid sequences (chosen by using the template HT[GC]EK[AP]) to retrieve sequences immediately before or after the selected sites). The four sequence logos were generated using WebLogo [15].

After applying the function *genomicLocToProteinSequence* of the R package to these 44 SUMO2/3 binding regions with the “SOX18-2bp-RBPJ1” composite motif, we obtained the protein sequences as well as the DNA sequences of the coding regions within these genomic sites. Figure 3C shows the logo graphs of the DNA sequences and protein sequences of all of the “SOX18-2bp-RBPJ1 motif” matching regions, using the on-line tool WebLogo [15] and Seq2Logo [16]. First note that the DNA motifs in Figure 3C are quite similar to the corresponding motifs in Figure 3A, but are more specific at some positions, because the former were obtained from a subset of the genomic regions from which the latter motifs were obtained. Comparing the DNA and protein sequences of the 44 SUMO2/3 binding regions in Figure 3C, it looks like the protein sequence is overall more conserved than the DNA sequence. However, at several positions the DNA sequence is more conserved. For example, the second amino acid in the protein sequence, Arginine(R), is coded by six codons in total according to the standard genetic coding scheme, for which Table 2 lists the (re-scaled) expected frequency of those six codons in human genome [17] and the (re-scaled) observed frequency of the six codons at the 44 genomic sites associated with SUMO binding. Table 3 compares the expected frequency and the observed frequency of the four codons coding the 9^th^ amino acid, Proline(P). In both cases, there is a large difference between the expected and observed frequencies of the codons coding the amino acid at one particular position in the 44 genomic sites. One specific codon appears in more than 90% of the 44 SUMO-associated sites and several other codons do not appear at all, while the expected frequency of all the codons coding the same amino acid is between 8 and 32%, indicating that the DNA sequences at those positions are more conserved than the corresponding amino acids. As a further test of conservation, we took advantage of the fact that the amino acid motif underlying the SUMO binding regions is repeated throughout the zinc finger regions of these proteins. We therefore compared the protein and DNA sequences of the surrounding N- and C-terminal sequence motif repeats and their codon usage bias. These adjacent motifs showed similar amino acid conservation (Figure 4B) but lower DNA sequence conservation (Figure 4B). This lack of DNA sequence conservation in the surrounding motifs is further emphasised by looking at the codon usage frequencies at diagnostic amino acid residues (Table 2 and Table 3). While clearly non-random, the highest usage of a codon was 62% rather than 90% found in the SUMO binding regions. Together these results therefore suggest that both the DNA sequences underlying the SUMO binding regions and the encoded protein sequences may have functional relevance.

**Table 2:** The expected and observed frequencies of the six codons for the 2^nd^ amino acid Arginine (R2) in the protein sequence in Figure 3C, and the observed frequencies of the six codons for R2 at the sites immediately before or after the 44 sites and with the similar amino acids in Figure 4D.^a^

	CGT	CGC	CGA	CGG	AGA	AGG
Expected frequency	7.9%	18.3%	10.9%	20.1%	21.5%	21.2%
Observed frequency at the 44 sites	0%	0%	4.7%	0%	93.0%	2.3%
Observed frequency at the sites immediately before or after the 44 sites and with the similar amino acids	0%	0%	15.3%	3.4%	69.4%	11.9%

^a^Expected and observed codon frequencies for R2 in the 44 SUMO-associated regions. Expected frequencies are calculated across the entire human proteome [17].

**Table 3:** The expected and observed frequencies of the six codons for the 9^th^ amino acid Proline (P9) in the protein sequence in Figure 3C, and the observed frequencies of the six codons for P9 at the sites immediately before or after the 44 sites and with the similar amino acids as in Figure 4D.^a^

	CCA	CCC	CCG	CCT
Expected frequency	27.7%	32.4%	11.3%	28.6%
Observed frequency at the 44 sites	0%	93.0%	7.0%	0%
Observed frequency at the sites immediately before or after the 44 sites and with the similar amino acids	10.2%	62.3%	2.9%	24.6%

^a^Expected and observed codon frequencies for P9 in the 44 SUMO-associated regions. Expected frequencies are calculated across the entire human proteome [17].

## 4 Discussion

We created an R package geno2proteo, a software dedicated to mapping sequences from any genomic and protein coordinates to reference DNA and protein sequences. We also created an online tool to allow the users to use the software directly from the web interface of the software. We illustrate how the package and online tool can be used to interrogate the protein and DNA sequences associated with genomic regions recovered by a ChIP-seq experiment. Here, it was initially ambiguous whether the DNA sequence conservation found under the SUMO binding peaks was a consequence of strong conservation of a protein coding sequence or rather was indicative of an underlying DNA motif that potentially acts as a protein binding site. Our analysis suggested that the latter is a possibility that warrants further testing in the future. We hope that the software will be useful in other studies involving genomic and proteomic data.
