# Supplementary material for: Geno2proteo, a Tool for Batch Retrieval of DNA and Protein Sequences from Any Genomic or Protein Regions
Source: J Integr Bioinform. 2019 Jul 13;16(3):20180090. doi: 10.1515/jib-2018-0090 (PMC6798850; doi:10.1515/jib-2018-0090)
Supplement: Supplementary file 1 [file jib-16-20180090-s001.zip › geno2proteoIntroduction(1).pdf]

An Introduction to the package *geno2proteo*

Yaoyong Li

March 15, 2019

Contents

1 Introduction **1**

2 The data files needed by the package *geno2proteo* **2**

3 The main functions of the package **3**

1 Introduction

In the bioinformatic area of mapping between genomic and proteomic data, it is often desired to find the protein sequences which are coded within a list of genomic regions specified by the coordinates on genome (e.g. "chr17 7668402 7673535 -"), and to find the genomic coordinates of a list of protein regions specified by the protein ID and the coordinates along the protein (e.g. "ENSP00000269305 281 391"). The package *geno2proteo* was created mainly to address the two basic tasks. It also provides the functions to find the DNA sequences of a list of genomic regions and the amino acid sequences of a list of protein regions.

In detail, this package tries to answer the following four questions which have been frequently asked in genomic and proteomic data processing:

- Given a list of genomic regions specified by their coordinates on genome, how can the protein sequences be obtained that are coded by these regions?
- Given a list of protein regions specified by their coordinates on the protein, how can the genomic coordinates of those protein regions be obtained from the corresponding genome.
- Given a list of genomic regions, how can the DNA sequences be obtained for these regions?
- Given a list of protein regions, how can the amino acid sequences of these protein regions be obtained.

The package provides four functions to try to answer the four questions.

The package needs three data files. One file is the genetic coding table. A file containing the standard genetic coding table is provided in this package. The other two data files are related to the particular genome and gene annotations that you want to use in your analysis. They can be conveniently downloaded from the ENSEMBL web site. A detailed explanation of those data files and the functions of the packages will be given in the following sections.

As some of the functions implemented in the package need to run some Perl scripts provided in this package, Perl needs to be installed in the computer in which you want to use this package.

The package *geno2proteo* is available at bioconductor.org and can be downloaded via `biocLite`.

```
> source("http://bioconductor.org/biocLite.R")
> biocLite("geno2proteo")
```

2 The data files needed by the package *geno2proteo*

The package needs three data files, which can be obtained quite easily, as explained as follows:

- **Genetic coding file**: A text file containing a genetic coding table of the codons and the amino acids coded by those codons. A file containing the standard genetic coding table is provided in this package, which is the file "geneticCode_standardTable_lines.txt" in the folder "geno2proteo/extdata/" which will be located in the folder that you install the package. This will be available after you have installed the package.

  Alternatively you can create your own genetic table if needed. The format of the genetic table used in this package is one line for one codon. The first column is the codon (namely 3 DNA letters) and the second column is the name (in a single letter) of the amino acid coded by the codon. You may have more columns if you want but only the first two columns are used by the package, and the columns are separated by a tab.

- **DNA sequence file**: A text file in FASTA format containing the DNA sequence for the genome that you want to use for analysing the data. The text file can be compressed by GNU Zip (e.g. gzip). You may download the file directly from the ENSEMBL web site. For downloading the latest version of genome from the ENSEMBL web site, you should follow the following steps.

  1. Go to the ENSEMBL data download web page, e.g. *http://www.ensembl.org/info/data/ftp/index.html/*
  2. Within the web page locate the species that you are interested in (e.g. Homo Sapiens), and then click the "DNA(FASTA)" column, and you will get another web page containing a list of files (for Home Sapiens the web page is *ftp://ftp.ensembl.org/pub/release-88/fasta/homo_sapiens/dna/*).
  3. Locate within the above web page the file name ending with ".dna.primary_assembly.fa.gz" (e.g. "Homo_sapiens.GRCh38.dna.primary_assembly.fa.gz") and download this file.

  For downloading the previous versions of ENSEMBL data, go to the ENSEMBL Archives web page

  http://www.ensembl.org/info/website/archives/index.html

- **Gene annotation file**: A text file in GTF format containing the gene annotations of the species that you are interested in. You may obtain this file from the ENSEMBL web site by following the three steps described above for obtaining the DNA sequence file, but with the following two modifications. One modification is in step 2: instead of clicking the column "DNA(FASTA)", click the "GTF" in the column "Gene sets". Another modification is in step 3: select the file name ending with ".<version>.gtf.gz" (e.g. "Homo_sapiens.GRCh38.90.gtf.gz").

Please make sure that the gene annotation file that you downloaded is from the same species and the same ENSEMBL version as the DNA sequence file.

Some of the functions in the package need another data file, which can be generated by a function in this package. In detail, once you have the three data files described above, you need to run the function `generatingCDSaaFile` in this package to generate another data file which will be needed by three of the four main functions in this package which have been mentioned in Section 1. This data file basically contains the genomic regions, DNA sequences and protein sequences of all of the coding regions (CDSs) specified in the gene annotation filem, which we may call as the *CDSs data file*.

The function `generatingCDSaaFile` has five parameters. Three of the parameters are the paths to the three data files that were described in the above. Another parameter is a folder to store the result file generated by the function. The last parameter is used to locate the executable Perl file in your computer which can be used to run Perl scripts. In detail, the parameters are

- `geneticCodeFile_line`: the name of the genetic code file.
- `gtfFile`: the name of the gene annotation file.
- `DNAfastaFile`: the name of the DNA sequence file.
- `outputFolder`: the folder where the result file will be stored. The default value is the current folder "./".
- `perlExec`: its value should be the full path of the executable file in your computer which can be used to run Perl scripts (e.g. "/usr/bin/perl" in a linux computer or "C:/Strawberry/perl/bin/perl" in a Windows computer). The default value is "perl"[1].

The function `generatingCDSaaFile` generates the CDSs data file which will be needed by some of other functions implemented in this package. The first part of the name of the data file is the same as the gene annotation file, and the last part is "_AAseq.txt.gz". Basically the data file contains all the coding regions, namely all the rows of *CDS* (coding DNA sequence) in the gene annotation file, and the DNA sequence and protein sequence of each CDS.

The following codes are an example of using the function `generatingCDSaaFile` to generate the CDSs data file by using the standard genetic code table, the first 3.5 million bases of the chromosome 16 in human genome (GRCh37), the ENSEMBL human gene annotations version 74 in this part of genome, the current folder as the the output folder, and the default value for "perlExec".

```
> library(geno2proteo)
> dataFolder = system.file("extdata", package="geno2proteo")
> geneticCodeFile = file.path(dataFolder, "geneticCode_standardTable_lines.txt")
> gtfFile_chr16 = file.path(dataFolder,
+ "Homo_sapiens.GRCh37.74_chromosome16_35Mlong.gtf.gz")
> DNAfastaFile_chr16 =  file.path(dataFolder,
+ "Homo_sapiens.GRCh37.74.dna.chromosome.16.fa_theFirst3p5M.txt.gz");
> tmpFolder = tempdir();
> generatingCDSaaFile(geneticCodeFile_line=geneticCodeFile,
+ gtfFile=gtfFile_chr16, DNAfastaFile=DNAfastaFile_chr16,
+ outputFolder=tmpFolder, perlExec="perl")
```

The resulting CDSs data file, "Homo_sapiens.GRCh37.74_chromosome16_35Mlong.gtf.gz_AAseq.txt.gz", contains all the CDSs and their amino acid sequences in the specified part of human genome and is in the current folder.

3 The main functions of the package

The package implements four functions to try to answer some basic and yet important questions in genomic and proteomic areas. Before describing those functions in detail, I would like to explain the formats that this package uses to define the genomic locus and the protein region, which are the input of these functions.

A genomic locus is represented by the name[2] and the strand of the chromosome where the locus is located, and the locus's start and end coordinates along the chromosome, in a specific genome. Note that the start coordinate is always less than or equal to the end coordinate. The strand of chromosome is denoted

[1]If the Perl executable file is in the computer's environment variable PATH and is available to R, which you can check by typing the command "perl -v" into your R interactive session to see if you can get the version information of the perl installed in your computer, you are safe to use the default value. Otherwise, you have to give the full path of the file in your computer which can be used to run Perl script.

[2]The chromosome name in the input data can be either in the ENSEMBL style, e.g. 1, 2, 3, . . . , and X, Y and MT, or in another popular style, namely chr1, chr2, chr3, . . . , and chrX, chrY and chrM. But they cannot be mixed in the input of one function call.

as “+” and “-” for forward and reverse strand, respectively. One example of genomic locus in human genome is “chr22 20127078 20127380 +”, which can also be represented as “22 20127078 20127380 +”.

A protein region, also called a location in this package, refers to a region in the protein consisting of the consecutive amino acids. It is represented by the ENSEMBL ID of either the protein or the corresponding transcript, and by the start and end coordinates along the protein. So the representation has two formats from which you can choose one in your convenience. For example, a region from the 91st amino acid to the 163rd amino acid in the protein corresponding to the transcript ZDHHC8-004 is represented as either “ENSP00000412807 91 163” or “ENST00000436518 91 163”, where ENSP00000412807 and ENST00000436518 are the ENSEMBL ID of the protein and the transcript ZDHHC8-004, respectively, in the ENSEMBL Homo sapiens GRCh37 V74 genome. Note that you can use any one of the two formats but have to use only one format in the input to one function call.

In the following I will describe in detail the four functions, and in particular the parameters and the output of each function.

1. `genomicLocsToProteinSequence` takes a list of genomic regions given in the input and tries to find the protein sequences and DNA sequences of the coding regions of genome which are within those genomic regions. It has two parameters:

   - `inputLoci`: a data frame containing the genomic regions as the input. Each row is for one genomic locus. The first column is for the chromosome name, the 2nd and 3rd columns are for the start and end coordinates of the locus in the chromosome, and the 4th column gives the strand of chromosome (“+” and “-” for forward and reverse strand, respectively) where the locus is in. Other columns are optional and will not be used by the function. Note that you can use any one of the two styles for chromosome name (namely either “chr1, chr2, . . . , chrX, chrY and chrM” or “1, 2, . . . , X, Y and MT”) but cannot use a mix of the two in the data frame.
   - `CDSaaFile`: the data file generated by the package’s function `generatingCDSaaFile`, described in the end of Section 2 .

   The function returns a data frame containing the original genomic regions specified in the input and after them, the five added columns:

   - Column “transId” lists the ENSEMBL IDs of the transcripts whose coding regions overlap with locus specified and the overlapping coding regions are exactly the same among those transcripts.
   - Column “dnaSeq” contains the dna sequence in the overlapping coding regions.
   - Column “dnaBefore” contains the DNA letters which are in the same codon as the first letter in the DNA sequence in the column “dnaSeq”.
   - Column “dnaAfter” contains the DNA letters which are in the same codon as the last letter in the DNA sequence in the previous column “dnaSeq”.
   - Column “pepSeq” contains the protein sequence translated from the DNA sequences in the three preceding columns, “dnaBefore”, “dnaSeq” and “dnaAfter”.

   The following codes illustrate the usage of the function `genomicLocsToProteinSequence` by using the CDSs data file generated by the codes in Section 2 and some genomic loci defined by their coordinates.

```
> library(geno2proteo)
> dataFolder = system.file("extdata", package="geno2proteo")
> # the input file
> inputFile_loci=file.path(dataFolder,
+     "transId_pfamDomainStartEnd_chr16_Zdomains_22examples_genomicPos.txt")
> #the CDSs data file
> CDSaaFile=file.path(dataFolder,
```

```
+     "Homo_sapiens.GRCh37.74_chromosome16_35Mlong.gtf.gz_AAseq.txt.gz")
> #the input data
> inputLoci = read.table(inputFile_loci, sep='\t', stringsAsFactors=FALSE,
+     header=TRUE)
> inputLoci = inputLoci[,1:4] # here just use the first 4 columns of input data.
> # But the input data can have as many extra columns as you like.
> proteinSeq = genomicLocsToProteinSequence(inputLoci=inputLoci,
+ CDSaaFile=CDSaaFile)
> # the 1st and 5th genomic loci in the input
> inputLoci[c(1,5),]

    chr   start     end strand
1 chr16 3166431 3166529      +
5 chr16 3169667 3169735      +

> # the DNA and protein sequences of the coding regions within the two loci
> proteinSeq[c(1,5),]

    chr   start     end strand
1 chr16 3166431 3166529      +
5 chr16 3169667 3169735      +
                                                         transId
1 ENST00000219091;ENST00000382192;ENST00000414351;ENST00000570935
5                ENST00000219091;ENST00000382192;ENST00000570935
                                                                                                  dnaSeq
1 CCAGTGACTTTCGAGGATGTGGCCTTGTACCTCTCCCGGGAGGAGTGGGGACGGCTGGACCACACGCAGCAGAACTTCTACAGGGATGTCCTGCAGAAG
5                              TACGCCTGCACTGACTGCGGGAAGCGCTTCGGCCGCAGCTCGCACCTCATCCAGCACCAGATCATCCAC
  dnaBefore dnaAfter                            pepSeq
1                    PVTFEDVALYLSREEWGRLDHTQQNFYRDVLQK
5                              YACTDCGKRFGRSSHLIQHQIIH

>
```

2. genomicLocsToWholeDNASequence takes a list of genomic regions and tries to find the DNA sequences of those genomic regions. Note that, as explained above, another function genomicLocsToProteinSequence returns the DNA sequences of the coding regions in the given genomic regions. In contrast, this function returns the whole DNA sequences of the genomic regions specified. This function has four parameters:

   - inputLoci: a data frame which has exactly the same format as the parameter "inputLoci" of the function genomicLocsToProteinSequence, which has been explained in the above.
   - DNAfastaFile: the name of the DNA sequence file for the whole genome, which has been described in Section 2 .
   - tempFolder: a temporary folder into which the program can write some temporary files which will be deleted when the function running is finished. The default value is the current folder.
   - perlExec: the same parameter as the one with the same name in the function generatingCDSaaFile, which has been described in Section 2 .

   The function returns a data frame containing the original genomic regions and after them, one added column for the DNA sequence of the corresponding genomic locus.

   The following codes illustrate the usage of the function genomicLocsToWholeDNASequence by using the CDSs data file generated by the codes in Section 2 and some genomic loci defined by their coordinates.

```
> library(geno2proteo)
> dataFolder = system.file("extdata", package="geno2proteo")
> inputFile_loci=file.path(dataFolder,
+     "transId_pfamDomainStartEnd_chr16_Zdomains_22examples_genomicPos.txt")
> DNAfastaFile =  file.path(dataFolder,
+     "Homo_sapiens.GRCh37.74.dna.chromosome.16.fa_theFirst3p5M.txt.gz");
> inputLoci = read.table(inputFile_loci, sep='\t', stringsAsFactors=FALSE,
+     header=TRUE)
> inputLoci = inputLoci[,1:4] # here just use the first 4 columns of input data.
> # But the input data can have as many extra columns as you like.
> tmpFolder = tempdir();
> DNASeqNow = genomicLocsToWholeDNASequence(inputLoci=inputLoci,
+     DNAfastaFile=DNAfastaFile, tempFolder=tmpFolder, perlExec="perl")
> inputLoci[c(1,5),] # the 1st and 5th genomic loci in the input

    chr   start     end strand
1 chr16 3166431 3166529      +
5 chr16 3169667 3169735      +

> DNASeqNow[c(1,5),] # the whole DNA sequences of the two loci

    chr   start     end strand
1 chr16 3166431 3166529      +
5 chr16 3169667 3169735      +
                                                                                                   DNAseq
1 CCAGTGACTTTCGAGGATGTGGCCTTGTACCTCTCCCGGGAGGAGTGGGGACGGCTGGACCACACGCAGCAGAACTTCTACAGGGATGTCCTGCAGAAG
5                               TACGCCTGCACTGACTGCGGGAAGCGCTTCGGCCGCAGCTCGCACCTCATCCAGCACCAGATCATCCAC

>
```

Note that the whole DNA sequences of the two loci shown in the above are the same as the DNA sequences of the coding regions within the two loci (as shown in the previous example), only because the two loci themselves are in the coding regions and do not contain any non-coding region. If a locus contains some non-coding regions, the whole DNA sequence of the locus will be different from the DNA sequence of the coding regions within the locus.

3. `proteinLocsToGenomic` obtains the genomic coordinates for a list of regions in proteins. The regions in proteins should be specified by the ENSEMBL ID of either the protein or the transcript encoding the protein and the start and end coordinates along the part of the protein. The function has two parameters:

   - `inputLoci`: a data frame containing the regions in the proteins as the input. The 1st column must be the ENSEMBL ID of either the protein or the transcript that encode the protein (or the equivalent of ENSEMBL ID if you have created your own gene annotation GTF file). But you have to use only one of two formats, and cannot use both of them, namely protein ID or transcript ID, in the input of one function call. The 2nd and 3rd columns give the coordinate of the start and end amino acids of the region along the protein sequence (namely the rank numbers of the start and end amino acids of the region on the protein sequence). Other columns are optional and will not be used by the function.
   - `CDSaaFile`: the same data file as the one used in the function `genomicLocsToProteinSequence`.

   The function returns a data frame containing the original protein regions specified in the input and before them, the six added columns for the genomic positions of the protein regions:

- The 1st, 2nd, 3rd and 4th columns give the chromosome name, start and end positions, and strand in the chromosome, which specify the genomic locus corresponding to the protein region.
- The 5th and 6th columns give the first and last exons in the context of coding exons in the transcript which correspond to the given protein region.

The following codes illustrate the usage of the function `proteinLocsToGenomic` by using the CDSs data file generated by the codes in Section 2 and some protein regions defined by their coordinates.

```
> library(geno2proteo)
> dataFolder = system.file("extdata", package="geno2proteo")
> # first using the ENSEMBL protein ID to specify the proteins
> inputFile_loci=file.path(dataFolder,
+ "transId_pfamDomainStartEnd_chr16_Zdomains_22examples_proteinID.txt")
> CDSaaFile=file.path(dataFolder,
+ "Homo_sapiens.GRCh37.74_chromosome16_35Mlong.gtf.gz_AAseq.txt.gz")
> inputLoci = read.table(inputFile_loci, sep='\t', stringsAsFactors=FALSE,
+ header=TRUE)
> genomicLoci = proteinLocsToGenomic(inputLoci=inputLoci, CDSaaFile=CDSaaFile)
> inputLoci[c(1,5),] # the 1st and 5th protein regions in the input data

        proteinID start end domainName geneSymbol PDBid          geneID
1 ENSP00000371627   123 155    PF01352     ZNF205    NA ENSG00000122386
5 ENSP00000460890   225 247    PF00096     ZNF205    NA ENSG00000122386

> genomicLoci[c(1,5),] # the genomic locations of these protein regions

    chr   start     end strand start_exon end_exon       proteinID
1 chr16 3166431 3166529      +     Exon_4   Exon_4 ENSP00000371627
5 chr16 3169667 3169735      +     Exon_4   Exon_4 ENSP00000460890
  start end domainName geneSymbol PDBid          geneID
1   123 155    PF01352     ZNF205    NA ENSG00000122386
5   225 247    PF00096     ZNF205    NA ENSG00000122386

> # then using the ENSEMBL transcript ID to specify the proteins
> inputFile_loci=file.path(dataFolder,
+ "transId_pfamDomainStartEnd_chr16_Zdomains_22examples.txt")
> inputLoci = read.table(inputFile_loci, sep='\t', stringsAsFactors=FALSE,
+ header=TRUE)
> genomicLoci = proteinLocsToGenomic(inputLoci=inputLoci, CDSaaFile=CDSaaFile)
> inputLoci[c(1,5),] # the 1st and 5th protein regions in the input data

          transID start end domainName geneSymbol PDBid          geneID
1 ENST00000382192   123 155    PF01352     ZNF205    NA ENSG00000122386
5 ENST00000570935   225 247    PF00096     ZNF205    NA ENSG00000122386

> genomicLoci[c(1,5),] # the genomic locations of these protein regions

    chr   start     end strand start_exon end_exon         transID
1 chr16 3166431 3166529      +     Exon_4   Exon_4 ENST00000382192
5 chr16 3169667 3169735      +     Exon_4   Exon_4 ENST00000570935
  start end domainName geneSymbol PDBid          geneID
1   123 155    PF01352     ZNF205    NA ENSG00000122386
5   225 247    PF00096     ZNF205    NA ENSG00000122386

>
```

As you can see, using either the protein ID or the transcript ID of the same protein regions generates the same results.

4. `proteinLocsToProteinSeq` obtains the protein sequences themselves for a list of regions in proteins specified by the coordinates of the regions along the proteins. It has the same two parameters as the function `proteinLocsToGenomic`, which has been explained above.

   The function returns a data frame containing the original protein regions specified in the input and after them, the added column for the amino acid sequences of the protein regions.

   The following codes illustrate the usage of the function `proteinLocsToProteinSeq` by using the CDSs data file generated by the codes in Section 2 and some protein regions defined by their coordinates.

```
> library(geno2proteo)
> dataFolder = system.file("extdata", package="geno2proteo")
> inputFile_loci=file.path(dataFolder,
+ "transId_pfamDomainStartEnd_chr16_Zdomains_22examples_proteinID.txt")
> CDSaaFile=file.path(dataFolder,
+ "Homo_sapiens.GRCh37.74_chromosome16_35Mlong.gtf.gz_AAseq.txt.gz")
> inputLoci = read.table(inputFile_loci, sep='\t', stringsAsFactors=FALSE,
+ header=TRUE)
> ProtSeqNow = proteinLocsToProteinSeq(inputLoci=inputLoci, CDSaaFile=CDSaaFile)
> inputLoci[c(1,5),] # the 1st and 5the protein regions in the input data

        proteinID start end domainName geneSymbol PDBid          geneID
1 ENSP00000371627   123 155    PF01352     ZNF205    NA ENSG00000122386
5 ENSP00000460890   225 247    PF00096     ZNF205    NA ENSG00000122386

> ProtSeqNow[c(1,5),] # the protein sequences of these protein regions

        proteinID start end domainName geneSymbol PDBid          geneID
1 ENSP00000371627   123 155    PF01352     ZNF205    NA ENSG00000122386
5 ENSP00000460890   225 247    PF00096     ZNF205    NA ENSG00000122386
                             pepSeq
1 PVTFEDVALYLSREEWGRLDHTQQNFYRDVLQK
5           YACTDCGKRFGRSSHLIQHQIIH

>
```
